# Regionally extended shared socioeconomic pathways for the offshore wind industry in Finland

**DOI:** 10.1007/s40974-022-00252-7

**Published:** 2022-06-16

**Authors:** Jamie Jenkins, Maria Malho, Kari Hyytiäinen

**Affiliations:** 1 Department of Economics and Management, University of Helsinki, 00014 Helsinki, Finland; 2 Demos Helsinki, Helsinki, Finland

**Keywords:** Scenario narratives, Renewable energy transition, Renewable energy, Participatory planning

## Abstract

Offshore wind energy is increasingly becoming an important part of European and global low-emission power systems. The aims of this paper are to create a shared understanding on the major drivers of offshore wind development in Finland and to explore how these drivers, and opportunities for the entire industry, may develop over the twenty-first century, under different global futures. This research develops extended shared socioeconomic pathway (SSP) narratives for the offshore wind industry by using a virtual participatory workshop with expert stakeholders. According to our results, the five key drivers shaping the prospects of offshore wind development are public acceptability of offshore energy, global and national demand for low-emission energy, technological development and relative competitiveness of offshore energy, availability of space and wind resources, and energy markets and transmission infrastructure. Nationally extended SSP narratives, building on these key drivers, describe a wide range of alternative future risks and opportunities for developing offshore energy. Under sustainable development (SSP1), offshore wind is likely to soon become a major source of energy in the area, if developed in a balanced manner alongside other uses of the marine space. Under fossil-fuelled development (SSP5), offshore wind grows slower and may experience rapid uptake only in the latter half of the century. Under the regional rivalry scenario (SSP3), the need for local energy sources drives the national energy policies and may create new opportunities for offshore wind. Under the inequality scenario (SSP4), local municipalities and the residents decide on locations of new wind turbines and the overall magnitude of future offshore wind.

**Supplementary Information:**

The online version contains supplementary material available at 10.1007/s40974-022-00252-7.

## Introduction

Renewable energy is an important tool for climate change mitigation. The planet is experiencing far-reaching and significant disruptions from the changing climate, and several policies and strategies are being implemented to curb the changing climate. The European Union (EU) has established policies aimed at increasing renewable energy supply, along with other climate change mitigating action. The EU has announced it has an objective of becoming an entirely carbon neutral continent by 2050. This European “Green Deal” introduces a roadmap for reaching the carbon neutrality target and outlines the needed investments and transitions. The EU has outlined plans to foster growth and investment in renewable energy and set targets for each EU member states to increase sources of renewable energy.

As of 2019, global offshore capacity is approximately 29.1 GW, and 80% of this is generated in Europe (Global Wind Energy 2020). Annual cumulative capacity of offshore wind in Europe has increased from 0.5 GW in 2010 to approximately 3.5 GW in 2019 (WindEurope 2020). Second only to Sweden, Finland is a leader in renewable energy production within the EU. Renewable energy comprises slightly over 40% of Finnish energy supply in 2018 (Official Statistics of 2020), mainly from combustible renewable energy sources such as biofuel. Offshore wind is a relatively underutilised source of energy in Finland, but with increasing demand for clean energy, offshore wind may become a competitive blue economy sector using coastal and marine space (Metsähallitus 2020). Finland is a sparsely populated country with relatively low land values, meaning most wind energy has been placed on land area, on coastal zones or forested hill tops. As of 2019, there are three connected offshore wind farms in Finland with a capacity of approximately 70.7 MW. This capacity level is expected to grow with development projects in varying stages. A maximum capacity of 2.7 GW of development projects is underway (Finnish Wind Power 2021). These wind farm projects will aid in achieving Finland’s renewable energy targets, and there is further untapped potential for offshore wind farms in Finnish waters.

There is a body of literature on sustainably balancing the use of marine space, including offshore wind development. Environmental concerns associated with offshore wind turbines include underwater noise from construction and operation impacting surrounding fish and marine mammals (Hüffmeier and Goldberg 2019), risk of collision for birds, potential habitat loss and displacement for marine mammals, birds, and other marine life (Soares-Ramos et al. 2020). Noise and visual issues can impact residents if built close to shore. Offshore wind farms are often highly costly to build given the specialised foundations, vessels, port and grid infrastructure and planning and maintenance required to commission offshore turbines (Bilgili 2011).

Finland has seen limited offshore development, but the potential is high, and demand is increasing. A similar situation is present in other parts of the world where potential for offshore energy is large, but development is slow. We should increase understanding on the opportunities and risks present in offshore development, and the compromises between the competing uses of the marine space (Virtanen et al. 2022). We are interested in understanding the drivers, risks and opportunities for offshore development under different global futures. This research also attempts to increase understanding on how to develop the Finnish marine space in a sustainable manner, while adjusting or reconciling the needs for clean energy alongside other uses of the marine space. Development should find a good compromise, or locally adjusted matches, between different uses of marine space. Scenario analyses exploring the consequences of alternative global futures on the drivers of offshore wind energy can be one way of exploring the risks and opportunities present in each sector. Scenario analyses can be a powerful tool to explore different research topics relevant for this paper, including complex multi-variable interactions (Spiecker and Weber 2014; Van Vuuren et al. 2010), socioeconomic changes (Victor 2012; Zhang et al. 2019) and different types of uncertainty under varying global conditions (Beiderbeck et al. 2021; Culot et al. 2020), among many other uses. Scenarios are well-established in climate change research, particularly climate change mitigation and adaptation research (O’Neill et al. 2020).

The shared socioeconomic pathways (SSP) are a recently developed scenario framework that supports climate change research. The SSPs represent 5 distinct, but equally plausible futures and describe future changes in socioeconomic conditions at the global scale over the twenty-first century (O’Neill et al. 2017). An emerging area of research is extending the SSP narratives to local, regional or industrial level (van Ruijven et al. 2014). A common approach is to use the global SSP narratives as boundary conditions and developing locally relevant scenarios through a stakeholder participatory approach. Such combinations of top-down and bottom-up approaches have been used in prior research to, for example, create locally relevant narratives for the Barents region (Nilsson et al. 2017), explore environmental changes in the Baltic Sea (Zandersen 2019), develop regional storylines for impacts, adaptation and vulnerability studies in the US Southeast (Absar and Preston 2015), study agricultural pathways in the US Northwest (Mu et al. 2019) and investigate climate impacts on river basins in Greece (Ker Rault et al. 2019).

The SSP framework is successful in supporting research across a varied range of research topics and has been used extensively across a range of scales, topics and methodologies (O’Neill et al. 2020). The framework has seen extensive use in energy research but has seen limited use in extensions to the Finnish energy sector. A prior study extends SSP-RCP framework to study the future wind conditions at the European level and focuses on certain areas, including Western Finland (Martinez and Iglesias 2021), but they do not interpret the SSP narratives in detail. In this research, we deepen prior knowledge and use existing methodological approaches. Global (O’Neill et al. 2017) and European (Kok et al. 2018) narratives are used as top-down, boundary conditions. Bauer et al. (2016) created the energy transformation pathways at the global scale for each of the SSPs. The European narratives are linked to the global narratives and assumptions for other important drivers at the global scale can be directly transferred from the global scale (Kok et al. 2018). Like the global scale narratives, the European energy pathways can be further extended to analysing a particular scale in more detail and act as regional boundary conditions.

The aim of this paper is to create a shared understanding on those drivers that impact the offshore wind industry in Finland and explore how offshore wind may develop over the twenty-first century, under different global futures. We are relying and building on earlier SSP literature and extending the Shared Socioeconomic Pathways (SSP) at the national scale to explore developments in one specific energy sector: offshore wind. We use a participatory stakeholder workshop as the bottom-up method to create locally relevant scenarios. Offshore wind is chosen as a case study as it shows potential to become a major renewable energy source in Finland, and Europe, but has challenges to overcome to reach this potential. We address a gap in understanding how and which drivers impact offshore wind development at a national scale and the relative importance of the drivers for the development of offshore wind. We also create narratives detailing how offshore wind may develop under four SSP scenarios. To our knowledge, this is the first study to extend this methodological approach for a specific energy sector at a regional or local scale.

## Materials and methods—co-creation of drivers and extended SSP narratives

We collected materials for extending the SSP narratives to Finland in a co-creative futures workshop (Jungk and Mullert 1987; Popper 2008) organised as a 1-day virtual event in January 2021. The aim was to use the co-creation method and stakeholder knowledge to understand better what the plausible SSP energy futures can mean for offshore energy at a national scale in Finland (Gudowsky and Sotoudeh 2017; Mauser et al. 2013). The underlying assumption and reason for organising the co-creation workshop was that collaboration with a diverse group of stakeholders and experts affects each directly and produces better outcomes than working alone (Austin and Seitanidi 2012a, b).

In November 2020, we invited 61 people to the workshop representing main stakeholder groups including entrepreneurs, officials of wind power associations, policy makers and public officers, researchers, regional administrators, spatial planners, energy industry representatives, military personnel and nature protectionists. The registered participants were provided with a report (supplementary material 1) introducing the scenario method in general, the SSP framework and a draft of plausible drivers for offshore wind in Finland based on a literature review. In the workshop, there were altogether 13 committed participants, 5 representatives from regional administrations and marine spatial planners, 1 from lobbying groups, 4 entrepreneurs, 1 researcher, 1 from nature conservation non-government organisation and 1 landowner. The workshop was organised virtually using an online meeting platform, enabling presentations and discussions with the whole group in a common room as well as working in smaller groups in separate breakout rooms. For documentation, we used a virtual platform to enable co-creative work online and in real time. We had prepared beforehand suitable canvases that included relevant background information as well as indicated spaces for documenting the group discussions in text boxes. Facilitators moderated each small group to support the discussion when needed and to help the participants in documentation and technicalities.

The workshop started with introductions and a virtual presentation of the introductory materials. After this, the first task given to the participants was to elaborate and rank drivers affecting offshore wind development in Finland, taking the introductory material as a starting point. We divided the participants into four breakout rooms and asked them to discuss with their group which drivers are relevant, how will they impact offshore wind, and if any drivers are missing. Next, the participants were asked to rank the drivers in terms of relative importance by placing them on a scale and expressing any causal relationships by arrows. The result collected in the canvas was a scale with a ranking of drivers affecting offshore wind development from most important to least important. Finally, each of the four groups presented their findings in a joint discussion in the common room, to share and reflect on the results together.

The second task of the workshop was to apply the drivers selected into the predetermined SSP scenarios to create scenario extensions for offshore wind development in Finland. Our objective was to explore and develop the extreme scenarios and explore the plausible consequences at the extreme end on the development of offshore. To this end, we decided to focus on SSP1, 3, 4 and 5 and not develop scenarios for SSP2, as this follows historical trends. We divided the participants into two small groups, which were each working with two SSP narratives. We asked the participants to fill in the relevant drivers for the development of the SSP in question in a table we had prepared beforehand in the canvas. The participants also documented in writing how the driver would develop towards the year 2100 in each of their two SSP scenarios. Moreover, they were also asked to evaluate and fill in the table the assumed impact of the driver on the competitiveness of offshore wind relative to other forms of energy production (e.g. nuclear, hydro, biofuels, solar, fossil fuels). The result of this working session was descriptions of the evolution of the most important drivers for each of the four SSP narratives. Before closing the workshop, the groups shared and reflected their group’s discussion and results in an open discussion.

## Results

### Drivers impacting offshore wind

**Fig. 1:**
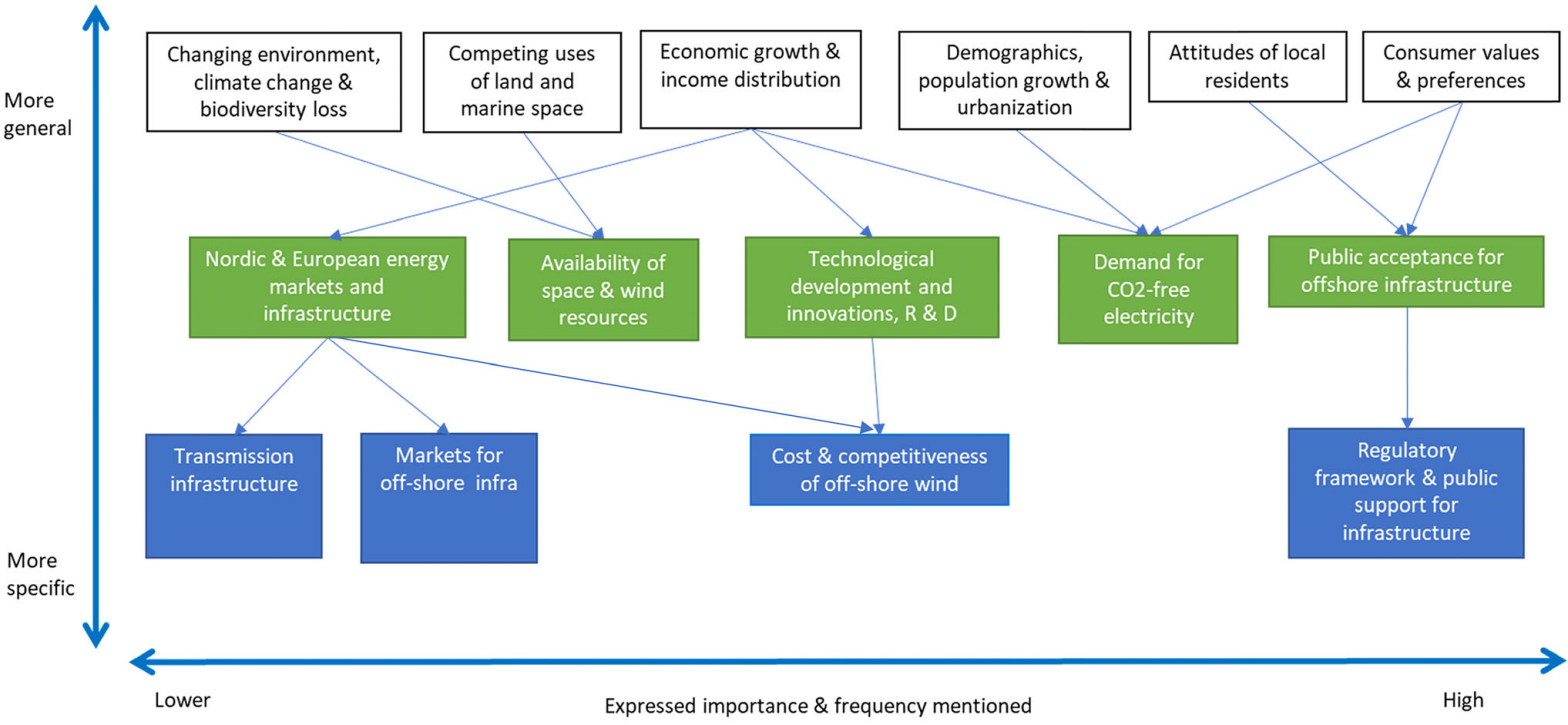
Main drivers impacting offshore wind development in Finland. The arrows represent the most important causal relationships between the drivers. The green boxes represent the key drivers of offshore wind energy identified by the participants. The higher-level drivers shape and impact these key drivers, while lower-level drivers follow from key drivers

Figure 1 summarises the main drivers of offshore wind energy and their interactions identified in the co-creative futures workshop. The *x*-axis represents the level of expressed importance and number of times mentioned in each discussion by the participants. The drivers that are higher on the *y*-axis represent fundamental, macro-level drivers. These background drivers are those that impact the overall economy, climate, markets, demographics and population. The next level down on the *y*-axis represents five key drivers that result from the background drivers. The final level of drivers on the *y*-axis is the secondary drivers. These drivers are trickle down results from the two higher-level drivers and directly impact offshore wind.

The first key driver of offshore energy is the *public acceptability for offshore infrastructure and* the *regulatory framework*. This provides the boundaries in which offshore wind can be developed. This is impacted by the attitudes of residents and, more broadly, the values and preferences of citizens through their voting power in municipalities and at the national level. In Finland, residents of the municipality where wind energy development is planned, and the landowners of neighbouring areas must be heard and have a say as part of the planning process. The values of citizens at a national level are also important as they can affect legislation. Groups of citizens can, and have made, initiatives for more stringent policies regarding offshore wind development. The political framework is further shaped through climate change and biodiversity loss as these drive consumer preferences and values. The regulatory framework and environment are influenced by the political will in an area and considerably impacts how, and if, offshore wind is developed. Supportive policies can greatly boost development.

The second key driver is the *demand for clean energy*. As a major driving force providing the incentives to develop renewable energy, demand for clean energy has trickle down impacts to offshore wind development. This demand is heavily driven by growth rates of the economy and population as well as the values and preferences of consumers. An increased number of people and an increase in economic growth and income lead to an overall increase in demand for energy. When consumer values and preferences shift toward renewable energy and away from traditional fossil fuel and other less-clean forms of energy, this boosts the demand for renewable energy sources.

Next, *technological development* is the third key driver. The triggering issue for the future success of offshore energy is how the cost-effectiveness of offshore energy develops in comparison with wind energy on land, and the costs of other low carbon ways to produce energy. Innovations in offshore wind technology may reduce the cost of transmission, storage and production. Adoption of emerging technologies (such as floating offshore turbines or use of hydrogen to store energy) that reduce the negative environmental impacts may also change consumer attitudes towards offshore energy. Public and private investment in research, development and innovation of offshore energy is critical for its future success.

As the fourth key driver the *availability of space and wind resources* can become a limiting factor, if competing uses of the marine spaces are considered more important than the development of offshore wind. In Finland, competing uses of the marine space include tourism, nature conservation, recreation, shipping and defence. The Finnish defence forces require full accessibility and possibility for surveying sea areas in the south of Finland. Due to this, the shallow seabeds of Bothnian Sea and Bothnian Bay are considered more suitable areas for development of offshore wind. Space allocated for energy in marine areas is further guided by marine spatial plans, which in turn is impacted by the acceptability of offshore energy by residents. Moreover, offshore farms are dependent on wind conditions being strong enough to generate enough power. Wind conditions may be influenced by the changing climate.

The final key drivers are the *energy markets and infrastructure*. An integrated and well-connected grid network and energy markets, nationally and between countries, are important for competitiveness and cost-effectiveness of producing, storing and supplying energy from offshore wind farms and distributing energy across networks. From an investor’s perspective, a well-established grid connection is a requirement for future investments into offshore wind development.

### Extended narratives for the offshore wind industry

The three levels of drivers identified (Fig. 1) serve as basis for extending the global SSP narratives to offshore energy at the national scale. Table 1 summarises findings from recently published research regarding how these drivers may evolve under each SSP narrative over the twenty-first century at the global and European scale. This information can be used together with the national narratives to provide global context on the development of offshore energy. This also allows us to compare the global-level trends with the extended and local trends in drivers important for offshore development and provides the boundary conditions.

**Table 1:** Summary of literature review and the resulting change in drivers in 4 of the SSP narratives

	SSP1: Sustainability	SSP3: Rivalry	SSP4: Inequality	SSP5: Fossil-fueled development
Demographics	Global population peaks around 2050 and decreases thereafter Urban population grows from 71 to 96%	Global population grows moderately and levels off 2070 Urban population grows from 71 to 91%	Global population grows fast in particular in developing countries and exceeds 12 billion in 2100 Urban population grows from 71 to 82%	Global population grows moderately and levels off by 2070 Urban population grows from 71 to 92%
Economic growth (Average world per capita GDP growth rate)	2.2% rate	1% rate	1.7% rate Economic growth is rapid in high income areas, but stagnant elsewhere	2.8% rate Rapid economic growth is coupled with high energy demand
Energy demand	Global energy production increases + 60% and peaks after 2070 Rapid electrification in rural and developing countries and rapid phase-out of fossil fuel-based sources Final energy demand increases slowly but is offset by improvements in energy efficiency and lifestyle changes Energy demand becomes decoupled to economic growth	Global energy production doubles by the end of the century High dependency on fossil fuels Slow electrification in developing countries increases demand for energy Carbon intensity remains high	Global energy production almost doubles by the end of the century High income areas see high energy demand and decreases in low to middle income regions High population growth in low-income areas increases energy demand Traditional biofuels remain important in low-income areas	Global energy production triples by the end of the century Heavy reliance on fossil fuel-based resources
Policy environment	Improved management of local and global issues, tighter regulation of pollutants Implementation of policies supporting the development of renewable energy and energy modernisation	Low priority for environmental issues Long term planning becomes rare Global cooperation is minimal, and governments are concerned with national and energy security	Environmental policies focus on local issues around middle- or high-income areas, little focus on vulnerable areas and global issues High income regions see strong government policies to tackle local environmental problems, but global issues are ignored	Environmental policies focus on local environments that have a direct, shown impact on human wellbeing, little concern for global issues Supportive policies are implemented for fossil fuel sources
Consumer preferences and local attitudes	Consumer preferences shift toward clean energy sources	Low public acceptability for renewable energy sources	Low consumer acceptance for conventional and non-conventional oil and gas	Low public acceptance for renewable energy sources
Political will to support renewable energy	Strong global institutions Europe sees high political stability and expansion. European governments are pressured to adopt ambitious measures for renewable energy transition	Weak short term policy ambition to mitigate environmental problem and limited renewable energy In Europe, the EU eventually breaks down with new regional blocs and alliances forming	Lack of global policy to reduce air pollution or promote renewable technology The EU is forced to find innovative solutions to natural resources depleting	High cooperation internationally for development issues but limited for environmental issues This hinders the transition to renewable energy sources, until fossil fuels become costly to extract. Overall, little support for renewable energy sources
Technological developments	Rapid advances in renewable energy technology Renewable energy becomes highly efficient and competitive Total factor productivity growth rate is 0.7% annually	Slow overall technological progress, particularly in energy efficiency Carbon capture storage and other emission reduction technology is slow to implement due to low technological improvements and high demand for land due to growing population Total factor productivity growth rate is 0.3% annually	Increased investment in high income areas leads to some improvements and cost reductions in different energy sources, both fossil fuel and renewable energy sources In Europe, technological developments do not lead to decreases in energy prices, due to monopolies controlling the energy supply Total factor productivity growth rate is 0.7% annually	Overall rapid improvements in technology, namely in the fossil fuel sector. Low improvements in the renewable energy sector Total factor productivity growth rate is 1.1% annually
Changing environment, climate change and biodiversity loss	Sharp declines in air pollution and greenhouse gas emissions	High levels of emissions, including from agriculture and emissions are constantly increasing High deforestation rates	An overall rise in pollution and emissions results from attempting to meet increasing energy demands Low-income areas increase non-fossil fuel emissions due to increasing agriculture to meet food demands and population growth. High global pollution and emissions High deforestation in low-income areas	High air pollution and emissions The environment degrades, but the population is unaware
Availability of space and competing uses of land	Food demand main driving force behind land use planning. By 2100, agricultural land is decreasing. Can impact development of onshore wind, thereby increasing competitiveness of offshore wind Land use is strongly regulated, and deforestation rates reduce In the OECD, land use is mostly pasture and crop	High demand for land due to growing population Large expansions of crop land Land use is hardly regulated	Land use is heavily regulated in high income areas	Medium regulations on land use High managed agriculture and high technological improvements in crop yields
Energy infrastructure	Existing fossil fuel infrastructure is gradually phased out which ultimately slows down the transition toward renewable energy However, production capacity for renewable energy sources is rapidly scaled up to meet demand and consumer preference shifts	Trade dependency is low, as does oil dependency. In Europe, regional conflicts and declining trade substantially increases food and energy prices nationally	In Europe, there is a large and widening gap between the high income, internationally connected societies, and lower income societies	Social acceptance for gas and fossil fuel infrastructure is high
Global markets and trade	Markets are generally well-connected and globalised International trade is moderate with strong regional production	High international trade barriers limit international trade and increases concern for national energy security	Trade between high income areas is free and open, restricted in other areas Markets are well-connected for the business and political elite, not connected for lower income	High international trade in food and energy and well-connected, globalised markets Significant exportation of coal
*Sources*	(Bauer et al. 2017; Dellink et al. 2017; Kok et al. 2018; Leimbach et al. 2017; O’Neill et al. 2017; Popp et al. 2017; Riahi et al. 2017; Samir and Lutz, 2017; Detlef P. van Vuuren et al. 2017)	(Bauer et al. 2017; Fujimori et al. 2017; Kok et al. 2018; Leimbach et al. 2017; O’Neill et al. 2017; Popp et al. 2017; Riahi et al. 2017; Samir and Lutz 2017)	(Bauer et al. 2017; Calvin et al. 2017; Kok et al. 2018; Leimbach et al. 2017; O’Neill et al. 2017; Popp et al. 2017; Riahi et al. 2017; Samir and Lutz 2017)	(Bauer et al. 2017; Kok et al. 2018; Kriegler et al. 2017; Leimbach et al. 2017; O’Neill et al. 2017; Popp et al. 2017; Riahi et al. 2017; Samir and Lutz 2017)

The second part of the workshop produced the regionally extended narratives for the Finnish offshore wind industry. The participants discussed how each driver would likely evolve over the twenty-first century and how this might impact offshore wind, and other forms of energy, under the 4 SSP narratives. The notes from the discussions were used to create the extended narratives, which are presented below.

#### SSP1—taking the green road

This scenario sees a shift toward renewable energy production. A stronger regulatory environment increases political will and changing consumer preferences toward greener energy and shifts the production to renewable energy sources. The political will and regulatory environment for developing offshore energy are strongly impacted by changing consumer preferences and values and the acceptance toward partially using marine space for offshore energy. This is a major driver of developments in the energy sector. This strong political will continue throughout the century and aids the development and implementation of renewable energy sources. Greater consideration is given to develop offshore energy so that the negative environmental impacts and damages to recreation possibilities are minimised. Offshore wind sees a moderate increase in competitiveness in comparison with other forms of renewable energy throughout the century and becomes an attractive source of energy unless unforeseen breakthroughs in other low-carbon energy technologies occur. Small-scale energy production and local solutions, including wind and solar power, become popular, especially as technology improves and becomes cost-effective. Technological development, coupled with EU and national policies supporting renewable energy, reduces cost of transmission, production and storage of offshore wind energy and other forms of renewable energy. For example, technological developments in floating offshore wind turbines and the ability to be located further from shore can reduce the environmental impact and provide greater flexibility for adjusting to competing uses of the marine space. Due to further advances in technology and cost reductions, wind farms are built further offshore further increasing the acceptability, attractiveness and competitiveness of offshore wind.

#### SSP3—a rocky road

This scenario is characterised by regional volatility and conflicts. International trade is restrained and there are high barriers for trading. Domestically in Finland, local township and municipalities see an increase in power as responsibilities are shifted from national governments to municipal governments. Restricted trade barriers lead to an increase in demand for national self-sufficiency in energy and raw materials and are a major characteristic and driving force behind the production and source of energy throughout the century. Cost determines which energy source and technology become preferred. At the beginning of the century, fossil fuels remain the cheapest source of energy. As reserves are depleted and become harder to extract, alternative and cheaper sources of energy are found. Wind power, particularly onshore wind, becomes competitive due to availability of technology, land space and capacity of energy production and self-sufficiency in Finland.

Globally, technological development is slow, and the same trend continues in Finland. Self-sufficiency requirements and concerns for national security means developments in technology are rapid in the agriculture and military sectors. As a requirement for undisturbed radar surveillance limits, development of offshore wind in Finland may be limited unless a common interest is found between wind power companies and the military. A common interest may include, for example, if military radar or sonar technology could be used in wind farms or if developments in military technology could be used in wind power infrastructure and development. Land ownership becomes an important status symbol and ownership is scattered between private owners. Scattered ownership makes it difficult to acquire land to develop onshore wind farms on a larger scale. However, this increases the development of small-scale wind farms on private land for self-sufficiency and increased revenue for landowners. Rural electrification increases throughout the century as the trend of urbanisation is slow, further increasing the demand for rural energy sources and small-scale energy production.

#### SSP4—a road divided

In this narrative, both globally and nationally, inequality is high. A select few in the political and business elite hold power. In Finland, municipalities have uneven interests in renewable energy, depending on local interest and wealth status. Those consumers that can afford to use renewable energy will, but the remaining population will use the cheapest available option. Revenue from taxation of offshore wind would dictate the interest and desire for placing wind turbines in the coastal regions of a municipality. Municipalities that are less well-off develop offshore wind to increase their tax revenue, whereas those areas that are more well-off reserve its coastal space for recreation activities, protection of coastal ecosystems and holiday housing. National monopolies may emerge in the energy market and can influence the development of technology and availability of different energy sources. These monopolies determine which energy sources are popular and available to consumers. This shift occurs depending on which industries these monopolies emerge and the company’s interest. Technological development is overall moderate throughout the century and the cost for energy storage, transmission and production overall decreases, but the deployment is less intense. Toward the mid-century, existing technology serves energy demand and is only upgraded and repaired as needed.

#### SSP5—taking the highway

This narrative sees rapid technological development and economic growth, low concern for environmental issues and high exploitation of fossil fuels. Global energy markets become highly connected and globalised. Finland becomes highly competitive in global markets due to rapid technological developments focussed on meeting high global energy demand, namely renewable energy technology and companies in this sector. Peat resources, the only fossil fuel resource in Finland, continue to be used and extracted throughout the narrative. However, peat may see decreasing use as local air pollution increases and the impact on health becomes increasingly known and widespread. This opens opportunities for new industries to develop and seize the opportunities that the high demand for energy creates. Renewable energy sources therefore become highly competitive, as does nuclear energy. New technologies and rapid progress are seen in the renewable energy sector, such as Power-to-X technologies and other carbon capture technologies. Sharp increases in urbanisation increase the need for electrification of area, particularly urban areas, and further boost energy demand. Wind power becomes a major source of energy in Finland, due to availability of space and technology and increasing energy demand throughout the century.

#### Summary of the prospects of offshore wind development

In SSP1, there are good prospects for offshore wind if it is developed in a balanced manner in line with other uses of the marine space. The negative externalities will need to be reduced with minimal harm to people and the environment. In SSP3, security concerns determine overall uses of marine space. Self-sufficiency and security of energy is a key driver on the source of energy. Offshore energy may be developed in-line with other energy sources nationally. The political environment and public acceptability in municipalities and local areas are key drivers of offshore development in the SSP4 narrative. Offshore wind energy has good prospects for areas that have limited other business opportunities. In some municipalities in Finland, the political environment may favour offshore wind if they lack other business opportunities, but development could be limited in areas that have a high number of holiday housing or recreational areas. In the final narrative, SSP5, the competitiveness of offshore energy compared to other sources of energy is the key driving force. Offshore energy may not be the most competitive in the beginning of the century but may gradually increase as other sources become fully exhausted, more costly or as technology improves. The political will and regulatory framework strongly limit the development of renewable energy, and regulation supports the continued use of fossil fuels.

## Discussion

This paper focuses on the important processes and factors that likely impact the development of offshore energy in Finland over a long-time span and detail alternative pathways on how offshore wind may develop over the twenty-first century. These pathways, extended for four global SSP scenarios, lead to quite different future outcomes in terms of the prospects for developing offshore energy. Narratives detailing offshore wind developments are limited, meaning there are few studies providing direct comparison. However, we can compare the general trends in energy and socioeconomic developments with prior narratives on energy issues at different scales. The global (O’Neill et al. 2017) and European (Kok et al. 2018)-level narratives were used as inputs to this study, and thus the main trends are mostly similar; however, there are some key details and nuances that create richer descriptions at the extended scale and reveal scenarios that are more relevant at the regional and national level.

SSP1 would likely see a competitive global offshore wind industry and rapid offshore upscaling globally and within Finland. Our extended SSP1 future largely follows that of the European and global developments in energy and social developments; renewable energy sees extensive development, green technology is prioritised, and governments implement ambitious measures to support the clean energy transition (Bauer et al. 2017; Kok et al. 2018).

The global SSP3 future reveals limited development of offshore energy and, more broadly, renewable energy. The cost-effective and locally available energy sources are used, which in most countries are fossil fuel-based sources. Wind power, as the space and technology are available, becomes relatively competitive in Finland as the extraction and importation of fossil fuel becomes costly and other energy sources are sought. In contract to the global scale, where renewable energy sees little development as cheaper fossil fuels are used and continue to be extracted to support increasing energy demand (Bauer et al. 2017).

In the extended SSP4 future, accepting offshore wind turbines may become a welcomed opportunity for increasing tax revenues for coastal municipalities in financial turmoil. Renewable energy may see development in less well-off Finnish municipalities to increase tax revenue, in contrast to global trends where cheaper fossil fuels are extensively used in less well-off areas (Bauer et al. 2017).

Finally, in SSP5, global offshore wind development sees limited upscaling in SSP5 until the end of the century. Fossil-fuel sources are preferred until these become costly. Renewable energy production becomes competitive early in Finland due to high energy demand and low-cost renewable energy options, in contrast to the global narratives where renewable energy is not competitive or widely implemented until later in the century (Bauer et al. 2017).

The first key driver of offshore development that we reveal in our results is public acceptance and a supportive political environment. Without public support, offshore wind is unlikely to see continued and expanded development in Finland. This is a sentiment echoed in previous research with prior studies finding community and public acceptance to be a key driver of development (Devine-Wright 2005; Jones and Richard Eiser 2010; Virtanen et al. 2022). Improving and managing public perception and acceptability of wind farm projects can help solve land-use and stakeholder conflicts at the local level, and increasing communication between planners and the public can achieve higher acceptance levels (Suškevičs et al. 2019). Involving the local community more directly in future land and marine space use is equally relevant for offshore energy and its future role as part of the energy system in Finland (Devine-Wright 2005; Nilsson et al. 2017). Furthermore, the EU has emphasised the need for stakeholder, industry and community engagement and discussion to improve sustainable development of offshore wind (Parliament 2022). The local community and stakeholders should be given the opportunity to express their views and opinions on offshore development projects in Finland and should be included in policy planning and discussions.

The second major driver revealed from our findings is the demand for clean energy. This energy demand is a critical force behind the development of offshore energy, which has also been shown to be a key component for how land is utilised and managed under different futures and has further impact on the type of energy produced (Rakovic et al. 2020).

The third and fifth key driving forces lead to similar consequences, as both technological innovations and a well-connected market and grid network increase the cost-effectiveness and competitiveness of offshore energy, mitigate the environmental impact, and improve public acceptability. Insufficient technological advances suited to a particular region can significantly limit development in that area. For example, offshore development in China was limited as technology was in its early stages and not suited to the local conditions (Liu et al. 2013) but has since boomed as technology has improved and competitiveness increased. In Finland, icy conditions are one factor limiting offshore development, and advances in technologies can increase the useable area for development. Furthermore, innovations in offshore technologies are strongly driven by a strong and supportive political environment. The burst of offshore development in the UK can be partly attributed to a coordinated effort from the UK government to boost renewable sources, and redirecting and channelling investment into offshore innovations and infrastructure (Kern et al. 2014).

The fourth main driver is the availability of space, particularly in the south of Finland, where offshore development is limited. One of the cited benefits of offshore wind power is the lower impact on competing uses of land, particularly when compared with its onshore counterpart (Bilgili 2011; Soares-Ramos et al. 2020). Onshore development has seen challenges in different regions, including northern Nordic regions with impacts cited on indigenous peoples (Normann 2021; Szpak 2019) and one-third of issues raised in environmental assessments of wind development in Canada relating to land-use planning (Dutta et al. 2021). Developing wind power offshore can partly alleviate some of these issues; however, offshore can also cause conflicts with other uses of the marine space and harm local ecosystems (Mooney et al. 2020; Pfeiffer et al. 2021; Voltaire et al. 2017). In Finland, spatial analyses have been undertaken to determine key locations for offshore development that minimise the impact to the surrounding environment and communities but are still economically viable (Virtanen et al. 2022). There is a need to simultaneously account for several issues present in Finnish waters: cost of transmission and building infrastructure, impacts on marine fauna and flora, needs of defence sector for surveillance and radar equipment, availability of shallow sea area far enough from the coast that limits visual impact on nearby households.

Our stakeholders find the change in wind conditions resulting from the changing climate a potentially major driver of offshore development. A prior study used two of the SSP narratives as inputs in a multi-model project exploring wind energy resources and wind availability across Europe and found wind power availability and density will drastically increase over the twenty-first century in the magnitude of 35% in western Finland (Martinez and Iglesias 2021). This sharp increase in wind power availability may significantly increase the development of offshore wind and become an even more important driver as the raw potential and opportunity for harnessing wind power off the Finnish coast becomes greater. This rapid increase of wind power density in Finland complements and aligns with the trends in the SSP5 narrative revealed in our results, where renewable energy, and wind power, becomes highly competitive.

Our list of drivers is by no means exhaustive, and there are other drivers that impact the development of offshore wind but were not explicitly discussed in our results. For example, electrification was revealed to be more important in impacting other drivers. Although not explicitly mentioned in Fig. 1, electrification could be part of the shift in consumer’s consumption patterns, demand for low-carbon energy and energy innovations and technology. Electrification was also seen as an important and growing factor impacting changes in global energy policy (International Energy 2021; Schiffer et al. 2018) and driving the development of renewable energy and, by extension, offshore wind (Shell 2017).

This research was conducted during two major global events—the COVID-19 pandemic and the war in Ukraine. The impacts from COVID19 on offshore are yet to be shown but may have longer-term impacts from changing work and travel habits. The Ukraine crisis has had a large immediate impact on the energy market, and the long-term consequences on the speed and the direction of energy transition will remain to be seen. In this paper, we have only studied four different futures and now reality has shown major changes that have had an impact on offshore development and the broader energy sector.

Research information on the drivers of offshore energy and the plausible future pathways of the sector may serve several purposes. Firstly, the results on drivers and plausible futures can be used to support and contribute to policy decisions and balanced governance of marine space, including development of offshore wind regionally and nationally within Finland while considering alternate and sustainable uses of the marine space. This information can lead to better informed policies, decisions and actions that impact offshore wind development. Furthermore, the results can support marine spatial plans at the regional level by revealing how offshore wind may develop and the relevant drivers of change, while considering the impacts on alternate uses of the marine space, particularly when combined with other quantitative and spatial data (Virtanen et al. 2022). Finally, these results can be used as inputs for other extended cases in different industries and sectors. Energy sector developments are important for many other related sectors including transportation and agriculture (Lehtonen et al. 2021).

Secondly, the results can support stakeholders and experts in the industry by providing detailed information for business decisions, investments and other decision-making processes. By revealing the opportunities and challenges facing the industry, businesses and investors within this industry can use this information to better understand their actions and decisions for long-term planning. Furthermore, the narratives can be used to identify the future challenges and opportunities facing the energy sector and the mitigation options for overcoming these challenges. This information provides valuable information on how the drivers impacting offshore wind, and more broadly, the energy sector in Finland may develop and the biggest opportunities and risks facing offshore wind development.

Finally, we have provided list of drivers, elaborated causalities, and developed locally relevant scenario narratives. These results offer ideas for businesses and practitioners to make use of the potentially emerging business opportunities and help policy makers to make better informed long-run decisions to ensure a balanced development of offshore wind. However, we do not offer solutions for either businesses or policy makers. For this end, a consensus of the national goal should be created, and transition pathways and roadmaps should be developed. Research extending normative scenarios for offshore wind is a natural next step in research.

## Supplementary Information

Below is the link to the electronic supplementary material.

Supplementary file 1 (PDF 642kb)
