# Supplementary material for: Regionally extended shared socioeconomic pathways for the offshore wind industry in Finland
Source: Energy Ecol Environ. 2022 Jun 16;7(6):533–45. doi: 10.1007/s40974-022-00252-7 (PMC9201803; doi:10.1007/s40974-022-00252-7)
Supplement: Supplementary file 1 — (PDF 642kb) [file 40974_2022_252_MOESM1_ESM.pdf]

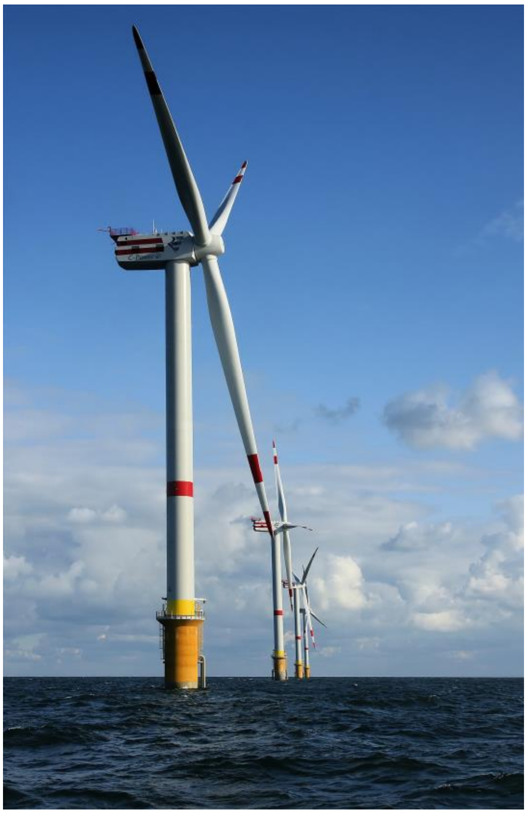

OFFSHORE WIND ENERGY 2100 - PATHWAYS FOR RENEWABLE ENERGY TRANSITION IN FINLAND

UNIVERSITY OF HELSINKI BlueAdapt

Introductory and preliminary material outlining the project information, methods, and background for the first workshop on the 14th January 2021.

UNIVERSITY OF HELSINKI, BLUEADAPT PROJECT

The workshop is part of BlueAdapt research project funded by the Strategic Research Council of Finland. The BlueAdapt project develops innovative means for supporting sustainable blue growth in food production, energy production and tourism.

You can get further information on the workshop and the project from:

Jamie Jenkins, Researcher, University of Helsinki: jamie.jenkins@helsinki.fi

Maria Malho, Expert, Demos Helsinki: maria.malho@demoshelsinki.fi

Kari Hyytiäinen, Professor, University of Helsinki: kari.hyytiainen@helsinki.fi

Introduction and Objective 2
What are scenarios? 3
What are Shared Socioeconomic Pathways (SSP)? 3
SSP narratives for the energy sector 5
SSP1 - Sustainability - taking the green road 5
SSP2 - Middle of the road 5
SSP3 - Regional rivalry - A rocky road 6
SSP4 - Inequality - A road divided 6
SSP5 - Fossil fuelled development - taking the highway 7
Numerical projections of SSP narratives 8
Drivers 11
Comparison of energy sources 12
Figure 6: Percentage share of energy consumption in Finland, 2020 12
Figure 7: Total wind power consumption (MWh) in Finland since 2010 13
Figure 8: Comparison of advantages and disadvantages of different energy sources 14
Prior energy scenario research 17
Next steps 20
Material and literature 21
References 22

OFFSHORE WIND ENERGY 2100 - PATHWAYS FOR RENEWABLE ENERGY TRANSITION IN FINLAND

Introduction and Objective

Offshore wind is projected to become a major source of renewable energy in Finland, and globally, over the coming decades. Offshore wind has an array of advantages over its onshore counterpart, and other forms of renewable energy. Advantages include higher possible capacity factor compared with onshore wind due to stronger and more consistent winds, greater capacity for larger farms and bigger turbines and less issues with visual and noise pollution if offshore. Please see the below energy comparison sections for more details. Offshore wind development in Finland, however, has been relatively slow and there are still challenges to overcome for the industry. Long-term planning and decision making are needed now to ensure smooth and continued development of offshore wind over the ongoing century to reach a future that is most desirable.

This project will bring together experts from within the offshore wind industry to discuss the opportunities and challenges facing the industry over the next century. The workshops provide you with an opportunity to network, share ideas, discuss, and co-create with other experts, policy makers, and researchers in the field.

The objective of this project is to create shared understanding on what could be done now, and in the future, to ensure that we seize the opportunities that the changing global conditions create for the Finnish offshore wind power industry. We do this by conducting a series of thought experiments in which we imagine Finnish society under different global futures, develop shared long-term vision for the offshore energy, and develop a roadmap of actions that take us there. The exercise will help at identifying strategic decisions that are robust under alternative global futures. The results consist of chain of policy actions, investments and decisions that help the offshore wind industry to adapt and take advantage of changing global conditions and the opportunities that arise under different global futures.

This project builds upon prior research in this field by expanding the exploration time horizon over the ongoing century. The Shared Socioeconomic Pathways (SSP) are used, which explores 5 distinct, yet equally plausible futures. The SSP method is discussed in more detail below.

The main aim of the project is to create practical policy recommendations for the Finnish offshore wind industry and the policy makers to reach the most desirable future given any global scenarios. The SSPs are used to explore the distinct global future scenarios.

In the first workshop we identify and rank the most important drivers that impact the Finnish offshore wind industry and explore changes in these drivers under different global futures and the resulting impact on offshore wind. These discussions will then be used to create scenario narratives and pathways under distinct global futures.

The first step is to identify the drivers (discussed in detailed below) that impact offshore wind and the overall energy market in Finland. After identifying the drivers and then ranking them in order of importance, we will break off into smaller groups to discuss how these drivers will develop in each global scenario.

The groups will then begin discussing how the most important driver will change over the next century. After a discussion on the most important driver, the next most important driver will then be explored, and so on after that.

What are scenarios?

Scenario analyses are well-established tools in environmental change research, especially in climate change mitigation and adaptation research. Various governmental and advisory panels have used scenario modelling to project climate change and emission trajectories under different futures and global changes. Scenarios can offer a tool to assess and explore how changes in global or industrial level drivers can impact the area of interest, offshore wind in this case. Recently, a global framework has been developed that describe different global developments over the next century. These global pathways, the Shared Socioeconomic Pathways (SSP), are used in scenario modelling research to represent 5 distinct global futures describing changes in socioeconomic conditions over the 21st century.

Other scenario analyses have been used in Finland to research different topics including analysing sources of electricity for district heat under a 100% fossil fuel free scenario, exploring possible futures of different used of Finnish marine areas under different environmental and socioeconomic conditions, and exploring different scenarios and pathways for an 80% reduction in greenhouse gas emissions by 2050. Please see the below materials and literature section for more details and links for further reading and examples of scenario analyses.

What are Shared Socioeconomic Pathways (SSP)?

The Shared Socioeconomic Pathways (SSP) represent 5 distinct, but equally plausible futures. These pathways describe future changes in socioeconomic conditions at the global scale over the 21st century. These narratives incorporate qualitative descriptions of socioeconomic drivers with quantitative numerical projections on drivers such as population growth, urbanisation, economic growth, energy supply and so on. The SSPs are unique in that they attempt to describe global futures in terms of challenges for mitigation and adaptation to future developments. Figure 1 below shows the 5 narratives relative to these challenges. SSP1, 3, 4 and 5 describe the extreme conditions with respect to challenges to mitigation and adaptation and SSP2 represents the “middle ground” scenario in which global trends will continue as they are today.

An emerging area of research is extending the SSP scenarios and pathways to local, regional, or industrial level. Different approaches are being used to explore future changes in socioeconomic conditions while using the global SSP narratives as boundary, or top-down, conditions. This research project is using the global SSP scenarios as boundary conditions and extending the narratives to the Finnish offshore wind industry level. We will explore how changes in global and regional level socioeconomic conditions and drivers will impact the offshore wind industry, and what actions and policies are needed now to reach the desired future under each global future.

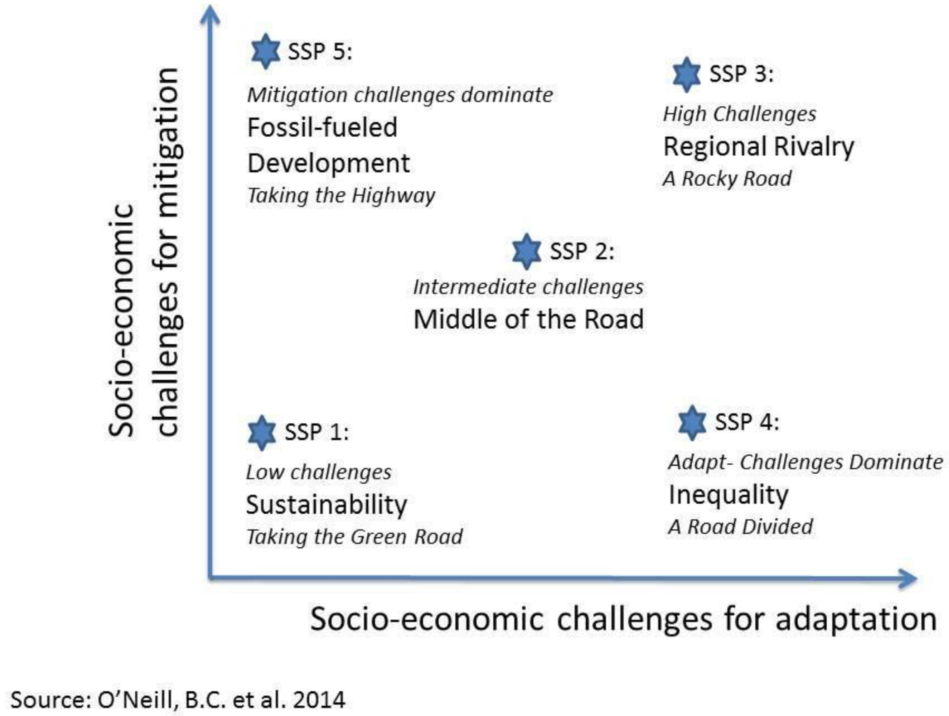

Figure 1. Five alternative visions for the year 2100.

SSP1 represents a future with low challenges to both mitigation and adaptation and describes a pathway of sustainable development and relatively high social welfare. On the opposite end, SSP3 is a future with high challenges to both mitigation and adaptation and is a future with high regional conflict, high nationalism, and closed borders. SSP5 is a future highly dependent on fossil fuel for energy sources, but has extremely high economic growth, low challenges to adaptation but high challenges to mitigation. SSP4 describes a pathway of high social and economic inequality, both globally and within a region. Challenges to mitigation are low, but adaptation challenges are high. The final scenario is the middle ground scenario, SSP2, that sees current global megatrends continue as they are now and has medium challenges to both adaptation and mitigation.

SSP narratives for the energy sector

The following section provides examples of each SSP narrative, with a focus on the energy sector. The changes in the global energy market are discussed first, followed briefly by the EU and Finnish level energy sector. These narratives are to provide an example on how different drivers might change under these different global futures. Please see the appendix section for additional detail.

SSP1 - Sustainability - taking the green road

Sustainable and inclusive development is the cornerstone of this pathway. Social, cultural, and economic costs of environmental degradation are better accounted for and management of environmental commons improves. Economic growth shifts toward an emphasis on human well-being which reduces inequality but may reduce growth in the short term. Technological development in the renewable energy sector is rapid and social acceptability for non-renewable energy sources is low. Carbon and energy intensity are low and consumption in material goods is low. Final energy use increases until the year 2060, with a flattening and eventual decline. Final energy demand increases slowly. Although rapid economic growth increases energy demand, this is compensated by rapid improvements in energy efficiency and lifestyle changes. Energy derived from wind power is variable throughout the projections but overall increases. Other non-biomass renewables drastically increase in the earlier 21st century and flatten around year 2090. Preferences shift away from fossil fuel-based energy sources and toward renewable and cleaner sources, including gas. Global markets are connected, and institutions are effective nationally and internationally. Environmental conditions begin to improve.

Population in the EU and within Finland gradually increases but flattens and begins to decrease near the end of the century. EU governments implement ambitious measures to stimulate an energy transition toward renewables and investments in health, education, and social support. International competition leads to greater innovation and research in renewable technology and by 2050, the EU is carbon neutral [1]. Economic growth gradually increases in both the EU and in Finland. Growth in the EU is the second highest in the narratives, and third highest in Finland.

SSP2 - Middle of the road

This road does not drastically shift from historical patterns in economic, social, and technological developments. Overall population growth is moderate. Global economic growth continues to grow and is slightly behind the SSP1 narrative, but growth continues unevenly between countries. Energy markets continue to grow at historical growth rates with medium improvements in energy technology. Energy mix does not shift from current trends and growth in the energy sector is moderate. Consumption continues to be materially intensive and carbon intensity is medium. Some developments are seen in the renewable energy sectors but the focus for technological development is on fossil fuels. Fossil fuel use remains prevalent, but energy sourced from wind power increases drastically and is the highest out of all the narratives. Other non-biomass renewable energy sources increase markedly throughout the projection and this narrative has the highest use at year 2100. Markets are semi-open and globalised. Global

policies lack focus on sustainability and only moderate success in implementing policies that attempt to combat environmental degradation. Environmental conditions continue to degrade.

The population in Finland continuously increases throughout the projection to reach a peak of just under 7 million in the year 2100. Finland's economic growth sees a gradual increase and is the second highest GDP out of the pathways. The EU population level peaks in year 2050, and then declines. EU economic growth gradually increases until year 2100 and is the third highest.

SSP3 - Regional rivalry - A rocky road

This pathway sees a fragmented world with rising nationalism and regional conflicts. Domestic issues are at the forefront of concern for policy makers so global commons and environmental degradation are poorly managed and lack political action. Countries focus on food and energy security for their own nation, at the expense of global economic growth and development. Resource and energy intensity are high and a heavy reliance on fossil fuels. Technological development is slow. National policies support the use of domestic fossil fuels and energy markets are limited. Globally, economic growth increases but slowly, and is the smallest out of the narratives. Energy use increases throughout the projections and is linked to economic growth. The use of non-biomass renewable energy sources is very limited and the smallest out of the projections. Energy sourced from wind power is severely lacking. There is serious environmental degradation.

The population level in Finland peaks in the year 2025, with a decline throughout the projection following this peak. The Finnish economy grows gradually until year 2080, followed by a flattening. This pathway has the lowest GDP in the year 2100. The EU level population and economic growth follows the same trend as the Finnish projections. The demand for energy within the EU, resulting from a gradual increase in the economy, creates the tipping point and leads to severe ecosystem failures. Eventually, a highly carbon-intensive, fragmented Europe emerges with large, energy intensive militaries. [1]

SSP4 - Inequality - A road divided

A pathway characterised by unequal economic growth, unequal investments in human capital and increasing inequality globally and within countries. Power is held by those in the political and business elite. Economic growth is overall moderate, but low in developing countries. In poorer countries, traditional fossil fuel-based energy is important. Consumption is high in this elite group, but the rest of society has relatively low consumption and low mobility. Technological development is high in oil and gas extraction, along with nuclear energy sources. Carbon intensity is low to medium. Constraints on fossil fuel use drives up prices. Non-biomass renewable energy sources gradually increase through the projection, but their usage does not drastically increase. Global institutions are effective for the elite groups, but ineffective elsewhere. Environment is generally well-managed in high income areas. The elite business groups are well-connected with global markets. The environment improves in high to medium income areas.

The Finnish population peaks in the year 2060 and declines thereafter. Economic growth in Finland continues a gradual increase until the year 2100. EU population is on a decline

throughout the projection. Economic growth flattens near year 2100, but gradually grows leading up to that point. The EU gradually increases its commitment to finding innovative solutions to depleting resources and a shift occurs toward green technology. However, inequalities lead to a select few companies controlling the energy supply, with no decreases in energy prices.

SSP5 - Fossil fuelled development - taking the highway

Economic growth is rapid but dependant on fossil fuels. Investment in education, health and human capital is high. Local environmental issues are addresses but global issues are not. Highly material lifestyles and high consumption. Fossil fuel use is extensive and energy demand is strongly linked to economic growth. Technological development is rapid in the fossil fuel sector and social acceptability for using these fuels is high. Global population increases until the year 2050, and then declines. Tourism and mobility are high. Global markets are connected, and policies focus on maintaining competitive advantage globally. Energy use sharply increases until 2080, and a flattening out follows this. Energy from non-biomass renewable sources remain low until year 2060 then see an exponential increase. The final level is higher than SSP1. This would presumably be due to a much higher population, higher economic growth, and a higher demand for energy. As traditional fossil fuel reserves begin to become harder to extract and the price increases, alternative energy sources are found. Wind power has very little uptake until year 2040, where a sharp increase occurs.

Both population and economic growth in Finland observe sharp increases. Population is almost twice that of the other narratives, and GDP is almost three times higher than SSP1 (the second highest GDP). The EU follows a similar pattern with a steady increase in population and a sharp increase in economics growth, with more than double the GDP at year 2100. Within Europe, a lack of environmental concern leads to abundant exploitation of fossil fuels. Europe experiences rapid economic growth, and part of this wealth is utilised for green technology innovation and research. Environmental degradation occurs while the population remains largely unaware and non-renewable resources are further exploited throughout the projection, leading to an increase in renewable technologies as non-renewable supply decreases [1].

Numerical projections of SSP narratives

Researchers have created a set of quantitative data to accompany and support the SSP narratives. This data quantifies changes in global drivers over the 21st century for each of the SSP narratives and includes drivers such as population growth, technological development, urbanisation and so on. This data is open source and available for all to view at from the International Institute for Applied Systems Analyses (IIASA), SSP public database website. Please see material section below for references.

The following section provides examples of the quantified drivers from the IIASA database. These are mainly in relation to energy supply and consumption, and global level drivers. These graphs numerically present the SSP narratives for different drivers and form the basis of the descriptive narratives above.

Figure 2: Global population

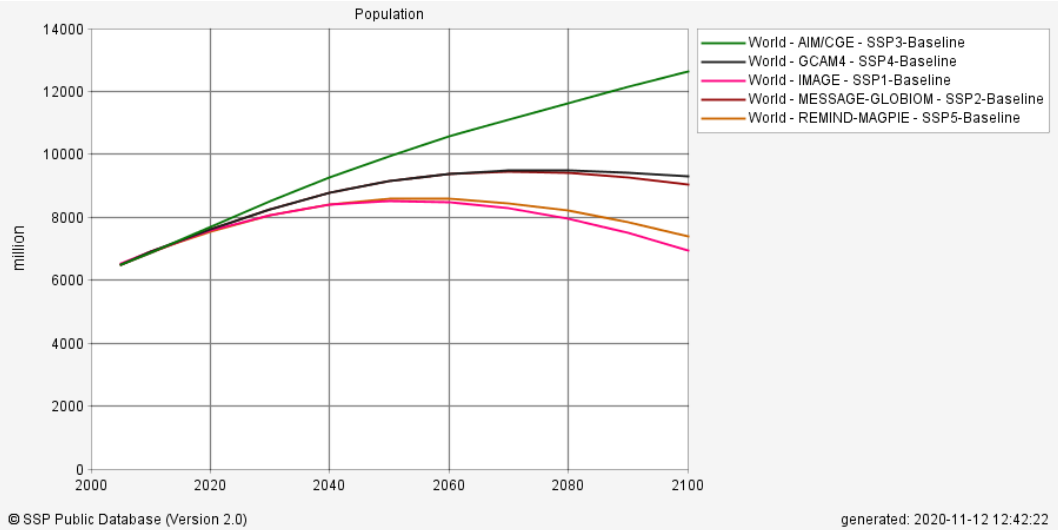

In all but scenario 3, population gradually increase up to the mid-century and starts declining thereafter. SSP 3 sees a gradual increase in population throughout.

Figure 3: Finnish population

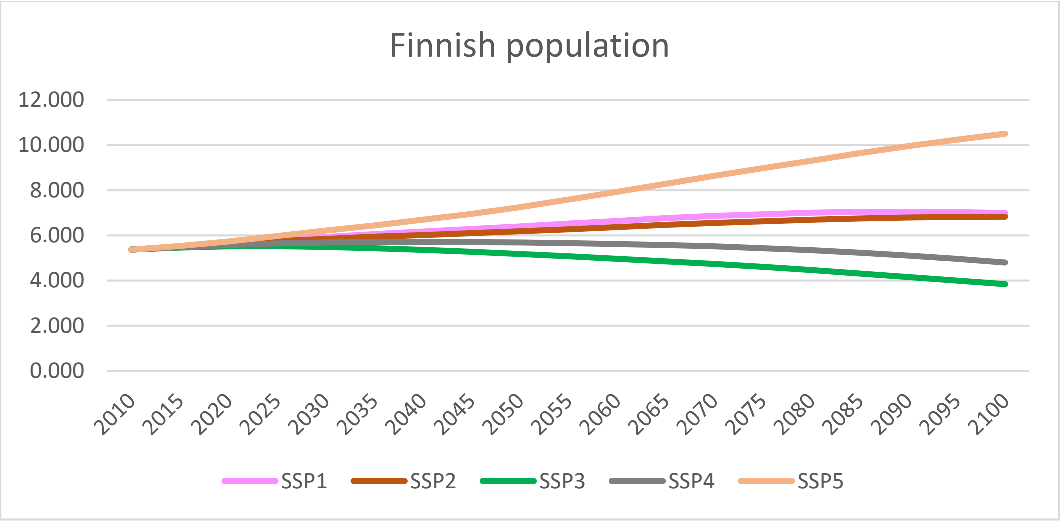

In most scenarios, the Finnish population level slowly declines except for SSP5 where the level almost doubles by year 2100.

Figure 4: Economic growth

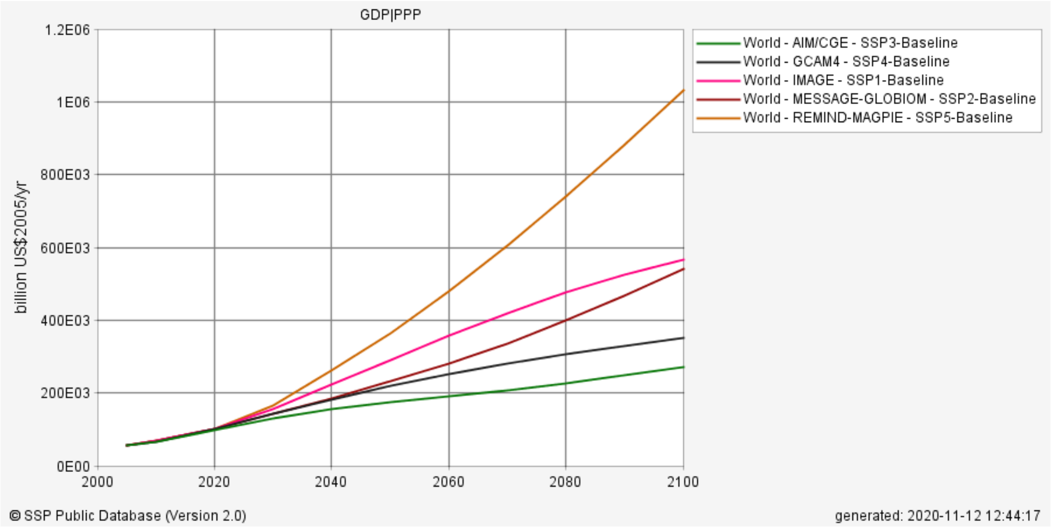

SSP5 has the most drastic increase in GDP, with the remaining scenarios showing a gradual increase. SSP1 has the second highest GDP at year 2100, but still significantly below SSP5.

Figures 5.1-5.4: Energy use

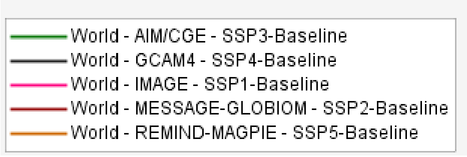

Figure 5.1: Total energy use

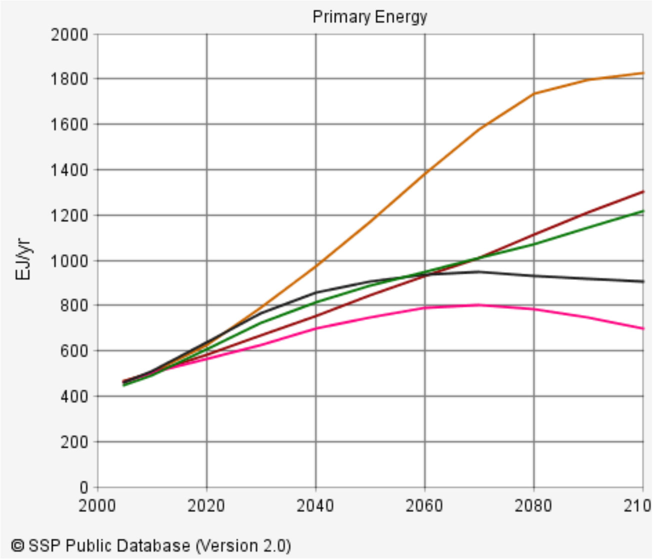

Figure 5.2: Wind (onshore and offshore)

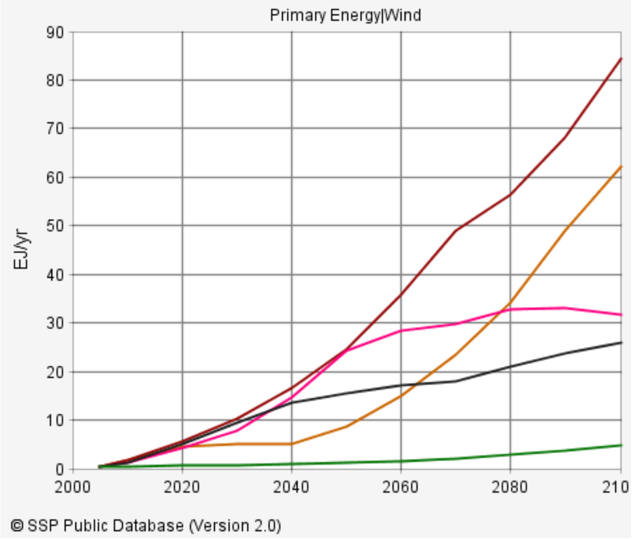

Figure 5.3: Non-biomass renewables

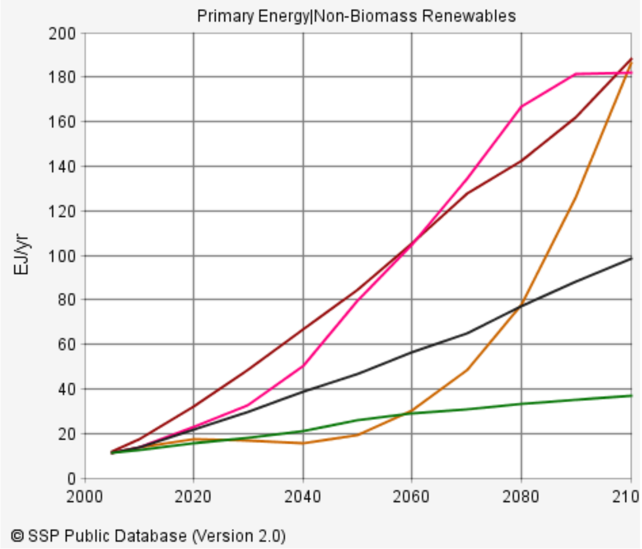

Figure 5.4: Fossil fuels

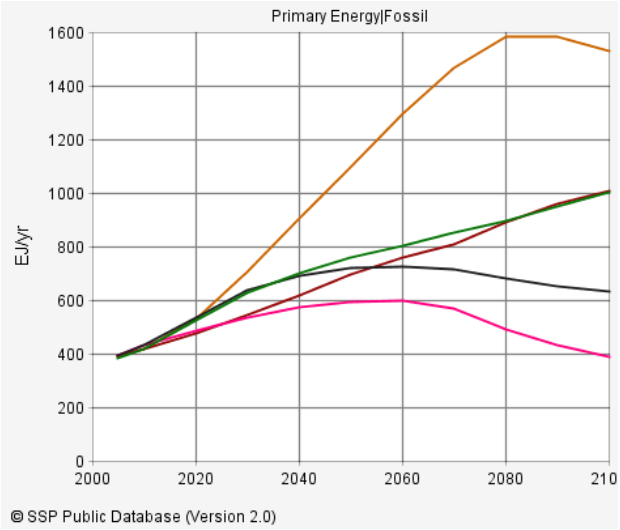

As the storylines above show, total energy use in SSP5 is drastically higher than the other scenarios. SSP1 has the lowest and a gradual decrease in total energy use. SSP5 also has the highest level of fossil fuel energy sources however around year 2080 fossil fuel reserves become limited and rapid development of renewable energy is shown. Energy use in SSP5 is great, so a drastic uptake in renewable energy is needed to maintain the same energy supply as fossil fuel sources become limited. Wind energy is gradual in SSP1, 3 and 4 but sees a sharp increase in SSP2 and 5.

Drivers

The forces of change, or drivers, are those phenomena or processes that have a significant impact on the offshore wind industry. Drivers can impact either the supply of resources for the industry or change or alter the demand for the products or service within the industry.

This section is to provide an example of those drivers that may impact the offshore wind industry in Finland. This is not an exhaustive list and there may be other drivers that impact the industry. Additional drivers can be chosen, or the existing list altered freely.

- Urbanisation and population distribution
- Population growth
- Economic growth
- Consumer preferences and environmental awareness
- Climate change and wind availability
- International relations and trade
- Policy and regulatory environment
- Competing use of land and marine space
- Technological development

Numbers of consumers, economic growth, income distribution as well as the lifestyles and preferences of consumers largely determine the aggregate demand for total energy. Population growth impacts the total demand for all energy. Climate change impacts wind availability. Economic growth partly defines the total demand for energy, depending on how much additional new household income is set aside for new energy. Consumer preferences guides the choice of which type of energy to consume, for example, whether to choose renewable energy sources or a cheaper alternative. Energy security and environmental awareness shift consumer preferences and development of infrastructure affects the ease and accessibility of developing new and cost-competitive energy sources.

Policy and regulatory environment impact the possibility of all energy industries to develop, including offshore wind. Competing uses of land and marine space affect the potential supply of new energy sources and in the case of offshore wind, can include tourism, military, nature conservation and so on. Wind availability directly impacts the capacity and overall supply of wind energy production.

Technological development impacts offshore wind and all other areas of energy production and their competitiveness and has an impact on many other drivers.

Comparison of energy sources

The following section explores and identifies the advantages and disadvantages of the different renewable energy sources and provides information on energy sources in Finland.

Figure 6: Percentage share of energy consumption in Finland, 2020 [3]

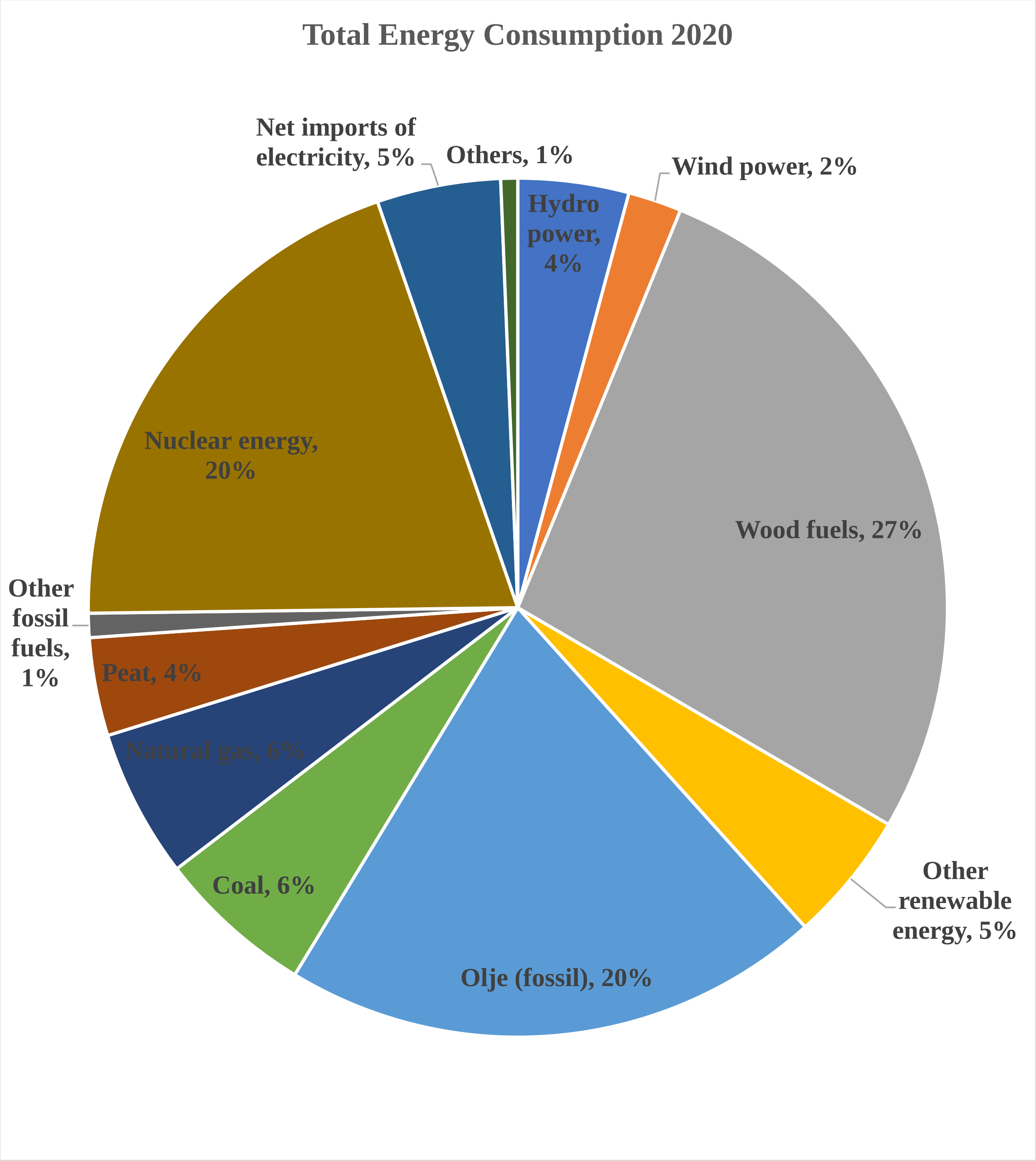

Non-renewable sources (other) still represent a substantial portion of energy consumption in Finland. This includes energy imports. Biofuels is the next large energy source, followed by nuclear. The remaining renewable energy sources make up a small portion of energy consumption.

Figure 7: Total wind power consumption (MWh) in Finland since 2010

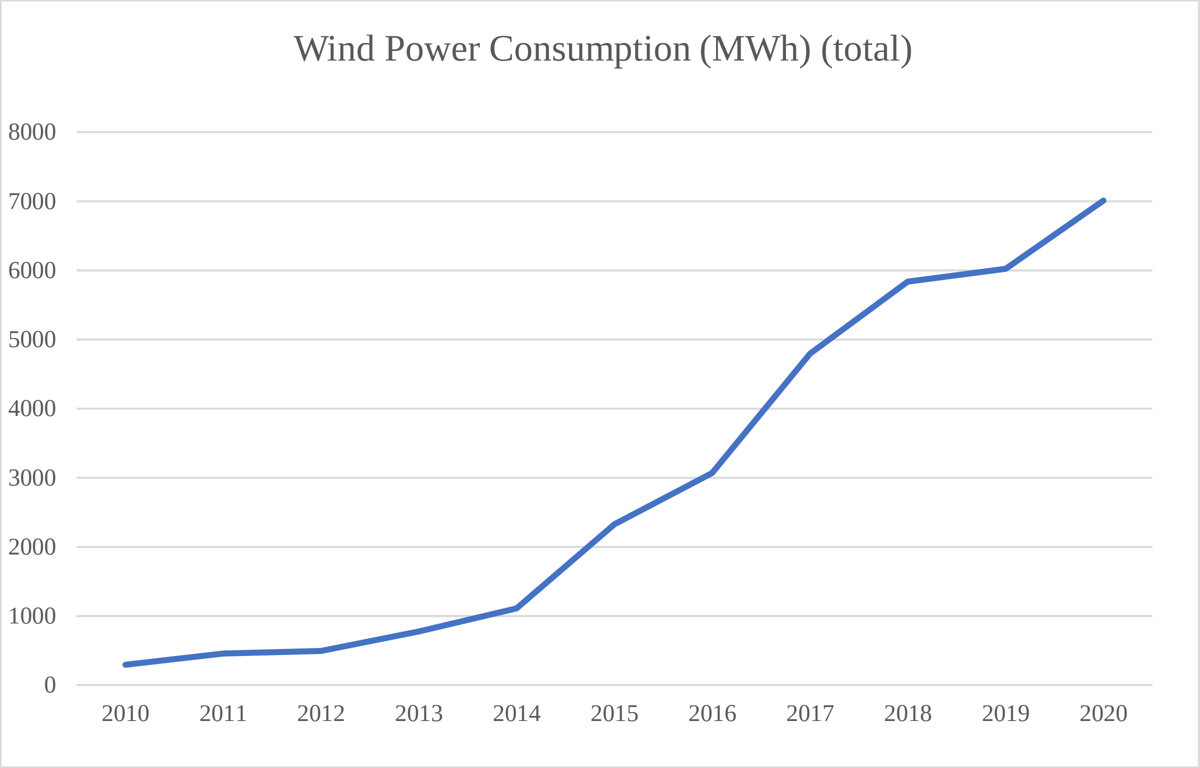

Consumption of winder power has seen a sharp increase since 2010. The majority of this capacity was from onshore wind power. Onshore wind power, at the end of 2019, was approximately 97% of total wind power capacity, with offshore wind accounting for the remaining 3%.

Figure 8: Comparison of advantages and disadvantages of different energy sources

The below table outlines a comparison of the advantages and disadvantages of renewable energy sources. This is not an exhaustive list and is open to interpretation.

	Offshore wind	Onshore wind	Hydropower	Solar power	Biofuels	Nuclear
Energy production	+ Capacity levels are higher compared with onshore as wind conditions are stronger and more consistent	– Can have lower capacity levels compared with offshore wind<br>– Can be unreliable as dependant on wind conditions	+ Generally high efficiency, capacity, and output [4]<br>+ Output can be regulated to match demand [4]	– Power generation can be intermittent and dependant on the sun being out	+ Can be a reliable source of renewable energy production and accounts for a large portion of energy production in some places already (eg. Finland)	+ Efficiency and reliability are greater than fossil fuel energy production [5]
Economic and profitability	+ Property taxes and income received to municipalities in Finland	+ Construction and maintenance costs are lower than offshore wind as conditions are not as difficult to install<br>+ Generally, cheaper to run compared with other energy sources [6]	+ Hydropower plants can be a long-time horizon investment and last a significant amount of time<br>– Expensive to install and build and very site specific [7]	+ Maintenance is easier as no moving parts [8]<br>– Battery technology is still expensive so storing energy can be expensive leading to energy only being generated in the sunlight hours	+ Usually, a side stream product of other industries (forestry) so adds additional profits to companies. But can be an expensive process to set up<br>+ Generally, inputs are low cost, and the supply is abundant [9]	– Expensive to build [10]<br>+ Relatively cost effective to run [11]

	Offshore wind	Onshore wind	Hydropower	Solar power	Biofuels	Nuclear
Environmental concerns	– Potential impact on marine mammals, seabirds, and other marine ecosystems [14]	– As with offshore wind, onshore wind can negatively impact local bird and bat wildlife. Infrastructure development around the wind farm can negatively impact local habitats and ecosystems	– Can have significant impacts on marine ecosystems and waterways [7]<br>– Can change temperature and flow of waterways [7]	+ No pollution to air or water [5]<br>– Production of the solar panels can cause significant emissions [8]	– Not a totally clean method of energy production as combustion is needed which releases emissions [15]<br>– Possible issue of deforestation if not sourced from a side stream [15] [9]<br>– Energy and heat are needed for the combustion process	– Nuclear waste disposal [11]<br>– Uses uranium to process energy which needs to be mined and is a finite resource [5]
Climate impacts	+ Very low to zero emissions from electricity generation from wind farms [16]	+ Very low to zero emissions from electricity generation from wind farms [16]	– Trapped vegetation can produce greenhouse gas emissions when they rot [6]<br>+ Does not directly pollute the water or air [5]	– No pollution or emissions during energy production from the solar panels<br>– Transportation and production can emit greenhouse gas and some toxic materials can be	– Can release emissions from combusting of organic materials [9]<br>– Resources are needed to grow and cultivate the crops to burn [15] [9]	+ Emissions are generally very low [5]<br>– Requires high levels of water for running of the plants [5]

				used in production [17]		
	**Offshore wind**	**Onshore wind**	**Hydropower**	**Solar power**	**Biofuels**	**Nuclear**
Competing land uses	– Military use – wind farms can interfere with military areas in the sea and radar technology used by militaries.<br>– Transportation and cruises in the Baltic – less of an issue in the Gulf/Bothnian Sea.	– Can compete with many different lands uses but depends entirely on area/region of wind farm.<br>+ Onshore wind farms can integrate with other land used such as agriculture	– Hydropower plants and dams can seriously impact surrounding land and sea uses with changes to waterways, creation of lakes, flooding and so on.	+ No competing land uses if used on roofs<br>– Large-scale solar power farms can compete with other land uses	– If material is not sourced from side stream products, can compete with forestry, agriculture, and other important land uses [6]	– Nuclear plants can compete with agricultural space and other land use. Disasters can make surrounding land unliveable and unviable for humans

Prior energy scenario research

Scenario analyses and research have become important tools to explore how changes in global or regional level drivers can impact the research area. The following section outlines various prior scenario research and analysis with a focus on energy in Finland. The table provides a summary of the research, its critical points and how it relates to SSP scenarios and the methods used in this project.

Title	Author	Summary	Critical points	Comparison to SSP scenario	URL
**Clean district heating - how can it work? (Fossil fuel-free energy scenario for Finland)**	Rinne, Samuli et.al	• Analysis of electricity sources for district heat in Finland in a 100% fossil free scenario compared with current situation	• Comparison of BAU state in district heating against a 100% fossil fuel free scenario. 40% of energy consumption currently from fossil fuels.<br>• Describes different methods on how to achieve 100% fossil fuel free district heating, and challenges to overcome.	• Comparable to energy use in SSP scenarios, projection is most like SSP1 – reduction in fossil fuels and increase in renewable energy.<br>• Argues wind power needs to increase to match energy demand and to become 100% fossil fuel free. Wind power is projected to increase in most SSPs	http://smartenergytransition.fi/fi/suomen-energiajarjestelma-voi-olla-taysin-fossiilivapaa/<br><br>http://smartenergytransition.fi/wp-content/uploads/2019/04/Clean-DHC-discussion-paper_SET_2018.pdf
**Scenario for 2050: A profitable alternative to the renewable energy system for Finland**	Child, Michael and Breyer, Christian	• Numerical projects under different energy scenarios analysing if Finland could become fossil fuel free, and the costs involved by 2050.<br>• Analyses 8 scenarios plus the BAU case as a comparison with low, medium and new nuclear energy and a low biomass case	• A 100% fossil fuel free scenario by 2005 is a highly competitive cost solution, with increasing share of nuclear<br>• Wind power is needed to reach a 100% renewable energy scenario by 2050, as is a drastic increase in solar power	• The results show an increase in renewable energy like most SSP scenarios, excluding SSP3. The biggest difference is wind power in this BAU case is relatively small compared with SSP2<br>• This study is also a static analysis at year 2040, so pathways are not shown.	https://www.lut.fi/uutiset/-/asset_publisher/h33vOeufOQWn/content/skenaario-vuodelle-2050-uusiutuvan-energian-jarjestelmasta-kannattava-vaihtoehto-suomelle<br><br>https://www.lut.fi/documents/10633/70751/LUT-Vision-and-initial-feasibility-of-a-recarbonised-Finnish-

					energy-system-for-2050.pdf
**Low carbon Finland 2050: VTT clean energy technology strategies for society**	Sipilä, Kai et.al	• Modelling and exploration of 3 different scenarios and pathways for a 80% reduction in GHG emissions by 2050 in Finland. The 3 storylines represent different industrial and technological developments that may occur in Finland over the next 30 years.	3 quantitative scenarios analysing cost-efficient pathways for achieving 80% GHG reduction<br>• Energy production may become carbon free by 2050<br>• Finland could reduce emissions by up to 90% if renewable energy solutions are adopted	• These scenarios show that an increase in renewable energy can aid in mitigating climate change and reducing GHG. The scenarios show an increase in renewable energy technology	https://www.vttresearch.com/sites/default/files/pdf/visions/2012/V2.pdf
**Gasgrid Finland and the Finnish gas sector: Scenarios 2040**	Gasgrid	• 4 different scenarios exploring the potential futures of the Finnish gas market and development of narratives under different social and technological developments	• Identifies 3 key areas of uncertainties for the gas market in Finland: society and social environment, gas and energy market and gas transmission infrastructure and technologies	• This work is like the SSP narratives: exploring the future under different socioeconomic changes but only explores until 2040 and a heavy focus on the gas market.<br>• Scenario 1 is like SSP1 – Finland pursues sustainable development and green technologies. Scenario 3 is like SSP3 – regional and global conflicts	https://gasgrid.fi/wp-content/uploads/Gasgrid-Finland-and-Finnish-Gas-Scenarios-2040.pdf
**Marine Spatial planning: Scenarios for the future of the maritime area**		• 3 scenarios exploring the possible futures of marine spatial areas in Finland under different environmental and conditions until the year 2050	• Energy and marine use for energy production is included as a exploration point, along with transport, environment, defence and other marine uses.	• Similar style of scenario work as the SSP narratives – exploring the future under different socioeconomic changes<br>• The 3 scenarios have similarities to the SSP narratives. EG – Scenario 3 in this work explored power struggles and regional conflicts, like	https://meriskenaariot.info/merialuesuunnitelma/en/scenario-introduction/

				SSP3, but varies slightly as EU is still in power. Scenario 2 explores a more green and sustainable development future. Scenario 1 is like SSP5 – high energy intensity and strong economic growth.	
**Finland's energy system for 2030 as envisaged by expert stakeholders**	Toivanen, Pasi et.al [19]	• Explores the Finnish energy market and systems through expert stakeholder interviews and their views on the energy system in 2030	• Interviewed experts all aim for full decarbonisation of Finland's energy system, but each had different views on the pathway to reach this goal.<br>• The main differences between these pathways relate to the extent of their support in international or national energy markets and the role of state governments.	• This paper uses exploratory scenarios and expert interviews but only until 2030.<br>• Some elements similar to SSP, however this paper focusses on the impact on consumers and companies rather than general drivers.	https://doi-org.libproxy.helsinki.fi/10.1016/j.esr.2017.09.007

Next steps

In the first workshop on the 14th January 2021:

- We will discuss the drivers that impact the offshore wind industry in Finland. The list above provides a starting point, and this can be expanded upon and discussed.
- The drivers will be ranked according to their importance for offshore wind.
- In groups, we will discuss and explore the development of each driver over the 21st century and its impact on offshore wind, and energy production.

After the first workshop:

- Narratives for each scenario will be drafted from the group discussions.

In the next two workshops

- Identify the future vision for the industry over the 21st century, from the regional and national perspectives
- Create, develop, and discuss the pathways that include the policies, practices and investments needed to reach this vision

The robust elements from these transition pathways will be identified and form the basis for the regional and national policy recommendations.

Material and literature

The following two articles presents the conceptual framework and presents descriptive narratives for each pathway.

Article title	Author	Year	Summary	Reference
A new scenario framework for climate change research: the concept of shared socioeconomic pathways	O'Neill, Brian et.al	2014	Presents the conceptual framework for building the SSP narratives	[20]
The roads ahead: Narratives for shared socioeconomic pathways describing world futures in the 21st century	O'Neill, Brian et.al	2017	Presents the descriptive narrative for each SSP	[21]

The following literature goes into detail on quantifying and creating SSP narratives, starting with energy related SSP narratives and into general quantifications. These lists the assumptions used for the quantification.

Article title	Author	Year	Summary	Reference
Shared Socio-Economic Pathways of the Energy Sector – Quantifying the narratives	Bauer, Nico et.al	2016	Quantifies the 5 SSP narratives for the energy sectors	[22]
New European socio-economic scenarios for climate change research: operationalising concepts to extend the shared socio-economic pathways	Kok, Kasper et.al	2017	Operationalise and conceptualise use of the SSPs	[1]
The Shared Socioeconomic Pathways and their energy, land use, and greenhouse gas emissions implications: An overview	Riahi, Keywan et.al	2016	SSP narrative descriptions and energy use	[23]
Energy, land-use, and greenhouse gas emissions trajectories under a green growth paradigm	Van Vuuren, Detlef et.al	2016	Quantification of the SSP1 narrative.	[24]
The marker quantification of the Shared Socioeconomic Pathway 2: A middle-of-the-road scenario for the 21st century	Fricko, Oliver et.al	2016	Quantification of the SSP2 narrative	[25]
SSP3: AIM implementation of Shared Socioeconomic Pathways	Fujimori, Shinichiro et.al	2016	Quantification of the SSP3 narrative	[26]
The SSP4: A world of deepening inequality	Calvin, Katherine et.ak	2016	Quantification of the SSP4 narrative	[27]
Fossil-fuelled development (SSP5): An energy and resource intensive scenario for the 21st century	Kriegler, Elmar et.al	2016	Quantification of SSP5 and energy use	[28]

References

1. Kok, K.; Pedde, S.; Gramberger, M.; Harrison, P. A.; Holman, I. P. New European socio-economic scenarios for climate change research: operationalising concepts to extend the shared socio-economic pathways. *Reg Environ Change* **2018**, *19*, 643-654.

2. International Energy Agency Electricity generation by source. https://www.iea.org/data-and-statistics?country=FINLAND&fuel=Energy%20supply&indicator=ElecGenByFuel.

3. Tilastokeskus Energy supply and consumption. **2020**.

4. energy4me Hydropower. https://energy4me.org/learn-about-energy/energy-sources/hydropower/pros-and-cons/.

5. Vieira, S. Pros and Cons of 10 Types of Energy. https://www.aje.com/arc/energy-types-pros-cons/.

6. BBC UK Comparing renewable sources of energy. https://www.bbc.co.uk/bitesize/guides/z8k9v9q/revision/3.

7. Better World Solutions Tidal Wave Energy, Pros and Cons. https://www.betterworldsolutions.eu/tidal-wave-energy-pros-cons/.

8. Better World Solutions Solar Power Pros and Cons. https://www.betterworldsolutions.eu/solar-power-pros-cons/.

9. Better World Solutions Biomass Power, Pros and Cons. https://www.betterworldsolutions.eu/biomass-power-pros-cons/.

10. Power Technology Nuclear power: The pros and cons of the energy source. https://www.power-technology.com/features/nuclear-power-pros-cons/.

11. Conserve Energy Future Nuclear Energy Vs. Fossil Fuel. https://sciencing.com/about-6134607-nuclear-energy-vs--fossil-fuel.html.

12. Giaquinto, R. Advantages and Disadvantages of Hydroelectric Energy. https://www.greengeeks.com/blog/hydroelectric-energy/.

13. Cason, M.; Satishchandra, R. A Cost and Benefit, Case Study Analysis of Biofuels Systems . **2015**.

14. Better World Solutions Wind Energy Pros and Cons. https://www.betterworldsolutions.eu/how-does-wind-energy-work-including-pros-cons/.

15. Ecavo The Advantages and Disadvantages of Biomass Energy. https://ecavo.com/biomass-energy-advantages-disadvantages/.

16. US Energy Information Administration Wind explained: Wind energy and the environment. https://www.eia.gov/energyexplained/wind/wind-energy-and-the-environment.php.

17. Vourvoulias, A. Pros and Cons of Solar Energy. https://www.greenmatch.co.uk/blog/2014/08/5-advantages-and-5-disadvantages-of-solar-energy#pollution.

18. RenewablesFirst How much does a hydropower system cost to build? https://www.renewablesfirst.co.uk/hydropower/hydropower-learning-centre/how-much-do-hydropower-systems-cost-to-build/.

19. Toivanen, P.; Lehtonen, P.; Aalto, P.; Björkqvist, T.; Järventausta, P.; Kilpeläinen, S.; Kojo, M.; Mylläri, F. Finland's energy system for 2030 as envisaged by expert stakeholders. *Energy strategy reviews* **2017**, *18*, 150-156.

20. O'Neill, B. A new scenario framework for climate change research: the concept of shared socioeconomic pathways. *Clim. Change* **2014**, *122*, 387-400.

21. O'neill, B. C. The roads ahead: Narratives for shared socioeconomic pathways describing world futures in the 21st century. *Global Environ. Change* **2017**, *42*, 169-180.

22. Bauer, N.; Calvin, K.; Emmerling, J.; Fricko, O.; Fujimori, S.; Hilaire, J.; Eom, J.; Krey, V.; Kriegler, E.; Mouratiadou, I.; Sytze de Boer, H.; van den Berg, M.; Carrara, S.; Daioglou, V.; Drouet, L.; Edmonds, J. E.; Gernaat, D.; Havlik, P.; Johnson, N.; Klein, D.; Kyle, P.; Marangoni, G.; Masui, T.; Pietzcker, R. C.; Strubegger, M.; Wise, M.; Riahi, K.; van Vuuren, D. P. Shared Socio-Economic Pathways of the Energy Sector – Quantifying the Narratives. *Global Environ. Change* **2017**, *42*, 316-330.

23. Riahi, K.; van Vuuren, D. P.; Kriegler, E.; Edmonds, J.; O'Neill, B. C.; Fujimori, S.; Bauer, N.; Calvin, K.; Dellink, R.; Fricko, O.; Lutz, W.; Popp, A.; Cuaresma, J. C.; KC, S.; Leimbach, M.; Jiang, L.; Kram, T.; Rao, S.; Emmerling, J.; Ebi, K.; Hasegawa, T.; Havlik, P.; Humpenöder, F.; Da Silva, L. A.; Smith, S.; Stehfest, E.; Bosetti, V.; Eom, J.; Gernaat, D.; Masui, T.; Rogelj, J.; Strefler, J.; Drouet, L.; Krey, V.; Luderer, G.; Harmsen, M.; Takahashi, K.; Baumstark, L.; Doelman, J. C.; Kainuma, M.; Klimont, Z.; Marangoni, G.; Lotze-Campen, H.; Obersteiner, M.; Tabeau, A.; Tavoni, M. The Shared Socioeconomic Pathways and their energy, land use, and greenhouse gas emissions implications: An overview. *Global Environ. Change* **2017**, *42*, 153-168.

24. van Vuuren, D. P.; Stehfest, E.; Gernaat, David E. H. J.; Doelman, J. C.; van den Berg, M.; Harmsen, M.; de Boer, H. S.; Bouwman, L. F.; Daioglou, V.; Edelenbosch, O. Y.; Girod, B.; Kram, T.; Lassaletta, L.; Lucas, P. L.; van Meijl, H.; Müller, C.; van Ruijven, B. J.; van der Sluis, S.; Tabeau, A. Energy, land-use and greenhouse gas emissions trajectories under a green growth paradigm. *Global Environ. Change* **2017**, *42*, 237-250.

25. Fricko, O.; Havlik, P.; Rogelj, J.; Klimont, Z.; Gusti, M.; Johnson, N.; Kolp, P.; Strubegger, M.; Valin, H.; Amann, M.; Ermolieva, T.; Forsell, N.; Herrero, M.; Heyes, C.; Kindermann, G.; Krey, V.; McCollum, D. L.; Obersteiner, M.; Pachauri, S.; Rao, S.; Schmid, E.; Schoepp, W.; Riahi, K. The marker quantification of the Shared Socioeconomic Pathway 2: A middle-of-the-road scenario for the 21st century. *Global Environ. Change* **2017**, *42*, 251-267.

26. Fujimori, S.; Hasegawa, T.; Masui, T.; Takahashi, K.; Herran, D. S.; Dai, H.; Hijioka, Y.; Kainuma, M. SSP3: AIM implementation of Shared Socioeconomic Pathways. *Global Environ. Change* **2017**, *42*, 268-283.

27. Calvin, K.; Bond-Lamberty, B.; Clarke, L.; Edmonds, J.; Eom, J.; Hartin, C.; Kim, S.; Kyle, P.; Link, R.; Moss, R.; McJeon, H.; Patel, P.; Smith, S.; Waldhoff, S.; Wise, M. The SSP4: A world of deepening inequality. *Global Environ. Change* **2017**, *42*, 284-296.

28. Kriegler, E.; Bauer, N.; Popp, A.; Humpenöder, F.; Leimbach, M.; Strefler, J.; Baumstark, L.; Bodirsky, B. L.; Hilaire, J.; Klein, D.; Mouratiadou, I.; Weindl, I.; Bertram, C.; Dietrich, J.; Luderer, G.; Pehl, M.; Pietzcker, R.; Piontek, F.; Lotze-Campen, H.; Biewald, A.; Bonsch, M.;

Giannousakis, A.; Kreidenweis, U.; Müller, C.; Rolinski, S.; Schultes, A.; Schwanitz, J.; Stevanovic, M.; Calvin, K.; Emmerling, J.; Fujimori, S.; Edenhofer, O. Fossil-fueled development (SSP5): An energy and resource intensive scenario for the 21st century. *Global Environ. Change* **2017**, *42*, 297-315.

APPENDIX

Global drivers impacting the offshore wind industry in Finland and their pathways under each SSP

Driver (source)	SSP1 : Sustainability	SSP2 : Middle of the road	SSP3 : Rivalry	SSP4 : Inequality	SSP5 : Fossil-fuelled development
1. General global trends	Management of the commons improves persistently but slowly. Strong institutions. Gradual move towards less resource intensive lifestyles. Focus on human wellbeing and equity rather than economic growth.	Socio-economic trends consistent with typical patterns of historical experience. Continued uneven income growth and societal stratification. Slow progress toward sustainable development goals. Reduced intensity of resource and energy use.	International fragmentation leads to regional rivalry and protectionism. Countries focus on achieving energy and food security goals within their own regions. Weak global institutions. Growing resource intensity. Consumption is material intensive.	Rising inequality and stratification both across and within countries lead to pockets of extreme wealth and poverty. Low and highly unequal investment in education. Reduced middle class.	Resource and energy intensive lifestyles prevail. Human development goals will be reached. Local environmental problems are met efficiently through technological solutions. Lack of global environmental concern.
2. Population / urbanization	Global population peaks around 2050 and decreases thereafter. Population in the EU and within Finland gradually increases but flattens and begins to decrease near the end of the century. The share of urban population grows from 71 to 96%.	Global population grows moderately and levels off 2070. The population in Finland continuously increases throughout the projection to reach a peak of just under 7 million in the year 2100The share of urban population grows from 71 to 91%.	Global population grows fast in particular in developing countries and exceeds 12 billion in 2100. The population level in Finland peaks in the year 2025, with a decline throughout the projection following this peak. The share of urban population grows from 71 to 82%.	Global population grows moderately and levels off by 2070. The Finnish population peaks in the year 2060 and declines thereafter. The share of urban population grows from 71 to 92%.	Global population peaks around 2050, but fertility remains high in high income countries. Population in Finland observes a sharp increase. The share of urban population grows from 71 to 96%.
3. Economic growth	World per capita GDP grows at an average 2.2% rate. However, GDP growth rate declines over time. Economic growth	World per capita GDP grows at an average 2% rate. Finland's economic growth sees a gradual increase and is the second	World per capita GDP grows at an average 1% rate. The Finnish economy grows gradually until year 2080,	World per capita GDP grows at an average 1.7% rate. Economic growth is moderate in industrialized countries	World per capita GDP grows at an average 2.8% rate. Increasing faith in competitive markets, innovation and

	gradually increases in both the EU and in Finland. Growth in the EU is the second highest in the narratives, and third highest in Finland.	highest GDP out of the pathways.	followed by a flattening. This pathway has the lowest GDP in the year 2100	and low in low-income countries. Finland's economic growth flattens near year 2100, but gradually grows leading up to that point.	participatory societies lead to high economic growth in Finland and globally.
4. Technological development	Directed development of environmentally friendly technologies. Moderate technological progress: total factor productivity growth rate of a technology leader is 0.7% annually	Moderate technological development without fundamental breakthroughs. Moderate technological progress: total factor productivity growth rate of a technology leader is 0.7% annually	Investments in education and technological development decline. Low technological progress: Total factor productivity growth rate of a technology leader is 0.3% annually	Technology development is high in the high-tech economy and sectors. Moderate technological progress: total factor productivity growth rate of a technology leader is 0.7% annually	Rapid technological progress: total factor productivity growth rate of a technology leader is 1.1% annually
5. International trade	Moderate with strong regional production, globalized and open trade	Moderate, weak globalized trade	International trade is strongly constrained due to stringent trade barriers, regionalized trade.	Globalized for connected elites, restricted access for non-connected population groups.	High, increasingly integrated global markets, regional specialization in production
8. Energy production	Global energy production increases +60% and peaks after 2070. Energy production is predominantly based on renewables, focus on energy efficiency	Global energy production will grow 250% by the end of the century. Moderate growth of the energy sector. Fossil-fuel dominated energy mix with some investment in renewables.	Global energy production doubles by the end of the century. Heavy reliance on fossil fuels. Energy security concerns support the use of domestic coal.	Global energy production almost doubles by the end of the century. Increasing shares of renewables and low-carbon energy sources.	Global energy production triples by the end of the century. Energy supply is fossil fuel based, no energy efficiency focus
9. Environ-mental policies & regulation	Improved management of local and global issues, tighter regulation of pollutants.	Concern for local pollutants but only moderate success in implementation	Low priority for environmental issues	Environmental policies focus on local issues around middle- or high-income areas, little focus on vulnerable areas and global issues	Environmental policies focus on local environments that have a direct, shown impact on human wellbeing; little concern for global issues
